# Immune-inflammatory endotypes of chronic rhinosinusitis: from epithelial alarmins to personalized therapy

**DOI:** 10.3389/fimmu.2026.1746626

**Published:** 2026-07-17

**Authors:** Hanxiong Li, Longting Wang, Dingbo Li

**Affiliations:** 1 Department of Otolaryngology, Shenzhen Longgang Otolaryngology Hospital & Shenzhen Otolaryngology Research Institute, Shenzhen, Guangdong, China; 2 Second College of Clinical Medicine, Shanxi Medical University, Taiyuan, Shanxi, China

**Keywords:** chronic rhinosinusitis, epithelial-derived cytokines, ILC2, immune-inflammatory endotypes, personalized therapy, T helper cells

## Abstract

Chronic rhinosinusitis (CRS) is a prevalent and complex inflammatory disease within otolaryngology, traditionally classified into phenotypes based on the presence (CRSwNP) or absence (CRSsNP) of nasal polyps. However, these phenotypic classifications inadequately capture the underlying pathophysiological heterogeneity of CRS. Recently, immune-inflammatory endotyping has emerged as a pivotal approach to better characterize CRS by delineating distinct inflammatory pathways, primarily type 1, type 2, and type 3 immune responses, each driven by unique immune cells and cytokine profiles. Epithelial-derived cytokines such as thymic stromal lymphopoietin (TSLP), IL-33, and IL-25, alongside immune cells including group 2 innate lymphoid cells (ILC2), T helper 2 (Th2), and Th17 cells, play critical roles in modulating the immune landscape of CRS. Moreover, variations in immune responses among different populations highlight the disease’s heterogeneity and underscore the need for precise immunological characterization. This review comprehensively summarizes recent advances in the immune-inflammatory endotyping of CRS, elucidates the underlying immunopathogenic mechanisms, and discusses the clinical significance of these endotypes. By integrating current research findings, this article aims to provide a theoretical foundation for the development of personalized therapeutic strategies tailored to distinct immune-inflammatory profiles in CRS patients.

## 1 Introduction

Chronic rhinosinusitis (CRS) is a prevalent and heterogeneous inflammatory condition affecting the nasal cavity and paranasal sinuses, characterized by persistent mucosal inflammation lasting at least 12 weeks. Traditionally, CRS has been classified into two clinical phenotypes based on the presence or absence of nasal polyps: chronic rhinosinusitis with nasal polyps (CRSwNP) and chronic rhinosinusitis without nasal polyps (CRSsNP) (1, 2). The clinical manifestations of CRS are diverse, ranging from nasal obstruction, discharge, facial pain, to olfactory dysfunction, and the disease significantly impacts patients’ quality of life and healthcare systems globally (3, 4). However, this phenotypic classification, while simple and widely used, has notable limitations. It fails to fully capture the underlying pathophysiological mechanisms and heterogeneity observed among patients, which are critical for accurate diagnosis, prognosis, and targeted therapeutic interventions (5, 6).

The traditional phenotype-based approach, relying mainly on endoscopic findings such as nasal polyps, does not adequately reflect the complex immunological and molecular mechanisms driving disease progression. For instance, patients with CRSwNP often exhibit T2 inflammation characterized by eosinophilic infiltration and elevated levels of cytokines such asIL-4, IL-5, and IL-13, whereas CRSsNP may involve non-type 2 inflammatory pathways with neutrophilic predominance (6, 7). Moreover, the phenotypic classification does not account for overlap syndromes or mixed inflammatory patterns, which are increasingly recognized as important contributors to disease heterogeneity (8, 9). Consequently, the need for a more nuanced classification system that incorporates immunopathological mechanisms has become apparent.

In this context, the concept of immunological endotyping has emerged as a pivotal advancement in CRS research and clinical practice. Endotypes are defined by distinct pathophysiological mechanisms, particularly the nature of immune cell involvement and cytokine profiles, rather than solely by observable clinical features (5, 6). The major endotypes identified in CRS correspond to T2, T2, and T3 immune responses, each characterized by specific cytokine milieus and immune cell infiltrates. T2 endotype, the most extensively studied, is associated with eosinophilic inflammation, elevated IgE, and a Th2-skewed immune response, commonly seen in CRSwNP and often linked with comorbid asthma and aspirin-exacerbated respiratory disease (AERD) (10, 11). T1 and T3 endotypes are characterized by interferon-gamma-driven and IL-17-driven responses, respectively, and are often associated with neutrophilic inflammation and non-eosinophilic CRS (12, 13).

The shift towards immunological endotyping is driven by the recognition that CRS is not a single disease but a spectrum of disorders with varying immunopathology, which influences disease severity, recurrence risk, and response to treatment (14, 15). For example, eosinophilic CRS (ECRS), a subtype of T2 endotype, is linked to higher rates of nasal polyp recurrence after surgery, more severe olfactory dysfunction, and comorbid asthma, necessitating tailored therapeutic strategies (1, 16). The identification of endotypes facilitates the use of targeted biologic therapies such as monoclonal antibodies against IL-4, IL-5, and IgE, which have shown efficacy in patients with T2 inflammation (10, 17). Furthermore, endotyping aids in predicting surgical outcomes and the likelihood of disease recurrence, allowing for more personalized clinical management (18, 19).

Immunological endotyping also underscores the critical roles of various immune cells and cytokines in CRS pathogenesis. Eosinophils, mast cells, T-helper cells, and epithelial-derived cytokines such as thymic stromal lymphopoietin (TSLP), IL-25, and IL-33 orchestrate the inflammatory milieu, contributing to tissue remodeling, mucin hypersecretion, and barrier dysfunction (13, 20, 21). For instance, hypoxia-induced upregulation of hypoxia-inducible factor-1α (HIF-1α) promotes the expression of epithelial-derived cytokines, enhancing T2 immune responses in ECRS (22). Moreover, the interplay between innate and adaptive immunity, including the involvement of innate lymphoid cells and regulatory T cells, shapes the inflammatory landscape and influences disease progression (23, 24).

Geographical and ethnic differences further complicate the immunological landscape of CRS. Studies have demonstrated variations in endotype prevalence and disease severity between Western and Asian populations, with non-eosinophilic and neutrophilic endotypes being more common in certain Asian cohorts (12, 19). These differences highlight the importance of considering population-specific factors in CRS research and the development of regionally adapted diagnostic and therapeutic approaches.

In summary, the limitations of traditional phenotype-based classification of CRS have led to the development and adoption of immunological endotyping, which provides a more precise understanding of disease mechanisms and guides personalized treatment. This paradigm shift integrates clinical features with molecular and cellular profiles, enabling improved prognostication and the implementation of targeted therapies, ultimately enhancing patient outcomes in CRS. In this review, we specifically examine how epithelial barrier dysfunction and the release of epithelial alarmins—TSLP, IL-33, and IL-25—serve as upstream ‘switches’ that program T2, T2, and T3 immune-inflammatory endotypes in CRS. By linking environmental triggers to alarmin signaling, downstream immune-cell modules, and clinically actionable biomarkers, we aim to provide a coherent framework for personalized therapy selection and response monitoring in CRS.

## 2 Main body

**Figure 1:**
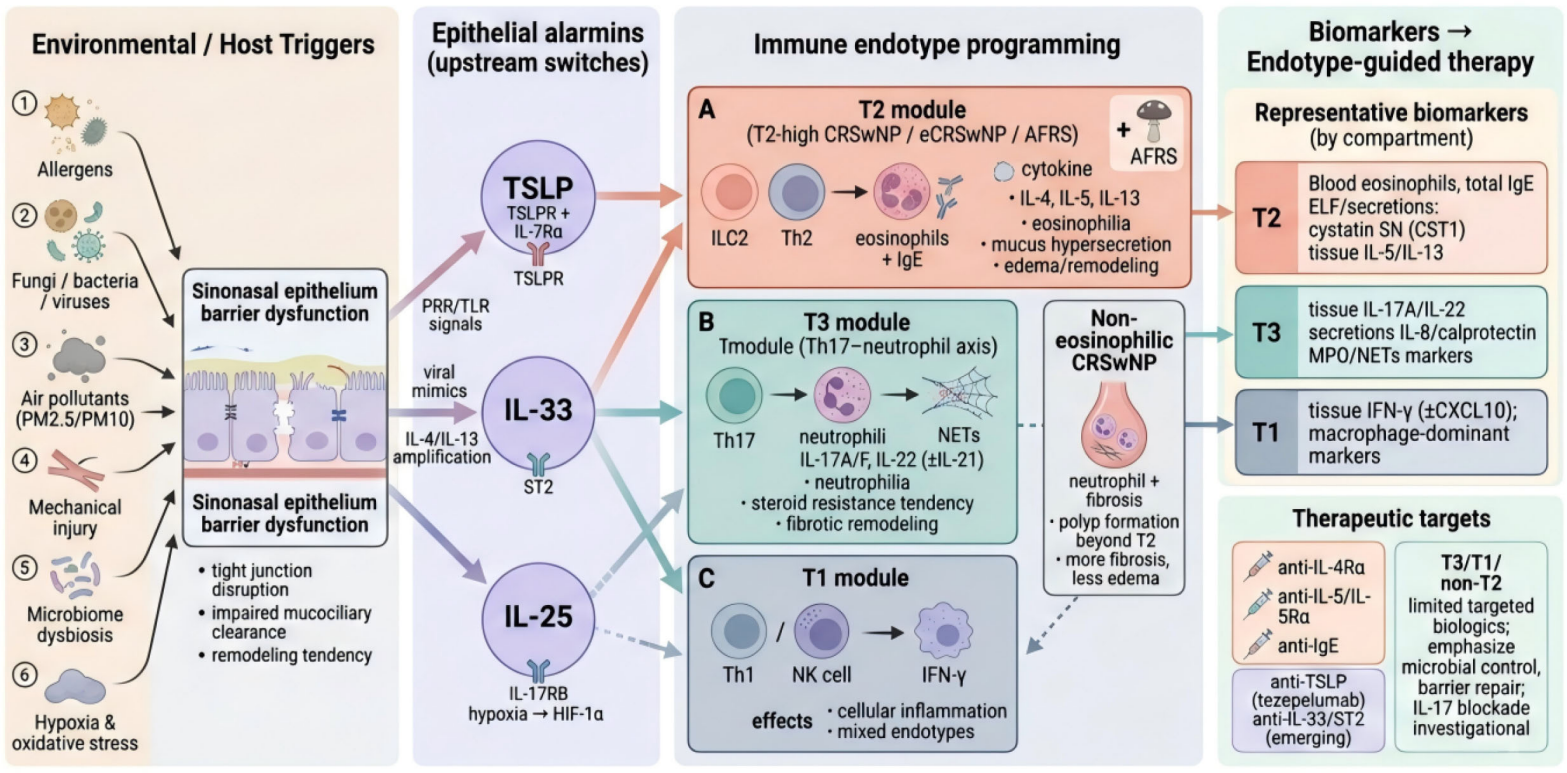
Epithelial–immune crosstalk programs CRS endotypes and informs precision therapy. Environmental and host-related triggers, including allergens, fungi, bacteria, viruses, airborne pollutants, mechanical injury, microbiome dysbiosis, hypoxia, and oxidative stress, disrupt the sinonasal epithelial barrier and impair tight-junction integrity and mucociliary clearance. Barrier dysfunction and pattern-recognition receptor signaling promote the release of epithelial-derived alarmins, particularly TSLP, IL-33, and IL-25, which act as upstream immune-programming signals. TSLP, IL-33, and IL-25 preferentially activate the type 2 module through ILC2s and Th2 cells, leading to IL-4, IL-5, and IL-13 production, eosinophilic inflammation, local IgE responses, mucus hypersecretion, edema, and tissue remodeling. Persistent microbial stimulation and dysbiosis may promote a type 3 module characterized by the Th17–neutrophil axis, IL-17A/IL-22 signaling, neutrophil extracellular trap formation, steroid resistance, and fibrotic remodeling. Type 1 inflammation is associated with Th1 and natural killer cell responses and IFN-γ-dominant cellular inflammation. Non-eosinophilic CRSwNP may arise from overlapping type 1 and type 3 pathways, with neutrophilic inflammation and fibrosis contributing to polyp formation beyond type 2 immunity. Representative biomarkers across tissue, epithelial lining fluid or nasal secretions, and blood may support endotype classification and treatment selection. T2-high disease may be treated with biologics targeting IL-4Rα, IL-5/IL-5Rα, or IgE, whereas upstream blockade of TSLP or IL-33/ST2 remains an emerging strategy.

CRS is initiated and sustained within a mucosal ecosystem in which the sinonasal epithelium functions not only as a physical barrier but also as an immunological sensor. Environmental insults (allergens, microbes, pollutants, and mechanical injury) disrupt epithelial integrity, promote oxidative stress and hypoxia, and trigger the release of epithelial-derived ‘alarmins’ such as TSLP, IL-33, and IL-25. These upstream cytokines subsequently shape distinct immune-inflammatory endotypes by preferentially activating specific innate and adaptive immune modules. Therefore, an epithelial-centric perspective provides a unifying entry point to understand CRS heterogeneity and to translate endotyping into personalized therapy (**Figure 1**).

### 2.1 Overview of immune inflammatory subtypes of CRS

The key immune-inflammatory endotypes, representative biomarkers, and therapeutic implications are summarized in **Table 1**.

**Table 1:** Immune-inflammatory endotypes in CRS: representative biomarkers across compartments and therapeutic implications.

CRS endotype (typical phenotype)	Core mechanism (epithelial → immune module)	Representative biomarkers – Tissue (biopsy/polyp)	Representative biomarkers – Secretions (ELF/NP swab/nasal lavage)
T2-high eosinophilic CRS (often CRSwNP, ECRSwNP; overlaps with asthma/AERD)	Barrier dysfunction + alarmins (TSLP/IL-33/IL-25) → ILC2/Th2 → IL-4/IL-5/IL-13; eosinophil activation, IgE class switching, edema/remodeling	IL-5, IL-13, IL-4; TSLP, IL-33, IL-25; eosinophil markers (ECP/EPX, PRG2/MBP); periostin; FcϵRI/IgE transcripts; epithelial CST1	CST1/cystatin SN in ELF (noninvasive T2 marker; correlates with CT/SNOT-22/eosinophils; AUC ~0.9); IL-5/IL-13 in mucus/lavage; ECP/EPX; periostin; local IgE; sometimes Charcot–Leyden crystal protein (galectin-10) signal
T2-low/”mixed” CRSwNP (polyp present but weaker T2 signature; may show overlap with T1/T3)	Partial alarmin activation + mixed downstream programming; co-activation of Th1/Th17 modules contributes to heterogeneity & variable steroid/biologic response	Modest IL-5/IL-13; mixed cytokines; variable eosinophils; TSLP/IL-33/IL-25 may still be elevated; co-expression of IFN-γ or IL-17A in subsets	Mixed mediator profile; lower CST1/IL-5 vs T2-high; possible elevation of neutrophil products (e.g., calprotectin) depending on dominant axis
AFRS (a T2-dominant fungal-driven allergic disease; often CRSwNP)	Fungal antigen exposure → strong T2 (IgE-mediated) + eosinophilic mucin; may show secondary Th17/neutrophil signals in some cases	Marked eosinophils; high IL-4/IL-5/IL-13; tissue IgE; alarmins (TSLP/IL-33/IL-25) may be increased; fungal-related inflammation signatures	Eosinophilic mucin components; local IgE; T2 cytokines; (optional) fungal sensitization markers not always from secretions
T3/Th17-neutrophilic CRS (classically CRSsNP, but also non-eosinophilic CRSwNP or mixed endotypes)	Microbial stimuli/barrier stress → IL-1/IL-6/IL-23 axis → Th17 → IL-17A/F, IL-22 → neutrophil chemotaxis, NETs; remodeling, steroid resistance in some cases	IL-17A/F, IL-22; IL-1β/IL-6/IL-23; neutrophil markers (MPO, ELANE); NET signatures; sometimes OSM; epithelial barrier/tight junction disruption markers	Neutrophil-derived calprotectin (S100A8/A9); IL-8/CXCL8; MPO/NET components (research use); elevated neutrophil chemokines
T1/Th1-dominant CRS (often CRSsNP; sometimes mixed)	Viral/bacterial triggers → IFN-γ/IL-12 axis → Th1/NK/CD8 activation; macrophage-dominant cellular inflammation; barrier dysfunction may be amplified by hypoxia	IFN-γ, IL-12; CXCL9/10/11; increased CD8/NK signatures; macrophage activation markers; epithelial PRR signaling upregulation	Type 1 interferon/chemokine patterns (research); less established secretion biomarkers for routine care
Non-eosinophilic CRSwNP (important in Asian cohorts; polyp with neutrophilic/fibrotic remodeling)	Polypogenesis via non-T2 modules: Th1/Th17 cytokines + neutrophils; fibroblast activation, fibrosis; epithelial tight junction disruption; OSM/TGF-β pathways	Mixed IFN-γ/IL-17A, IL-1β/IL-6; neutrophil markers (MPO), OSM; fibrosis markers (TGF-β, ECM remodeling signals); less edema, more fibrotic architecture	Calprotectin/IL-8 tendency; lower T2 mucus markers (CST1/IL-5) compared to T2-high
Epithelial-alarmin-high state (upstream “trigger” module) (cuts across phenotypes/endotypes)	Environmental insults + hypoxia/oxidative stress → epithelial release of TSLP, IL-33, IL-25 that programs downstream T1/T2/T3	Elevated TSLP/IL-33/IL-25 in epithelium; HIF-1α-linked signatures; barrier disruption markers (tight junction proteins downregulated)	Potential detectability of alarmins in secretions (variable, assay-dependent); could complement endotyping

#### 2.1.1 Type I inflammation and its immune characteristics

Type I inflammation in CRSis primarily characterized by a Th1-skewed immune response dominated by interferon-gamma (IFN-γ) and interleukin-12 (IL-12). This endotype is strongly associated with chronic rhinosinusitis without nasal polyps (CRSsNP) and is largely triggered by bacterial and viral infections. The hallmark cytokines IFN-γ and IL-12 orchestrate a cellular immune response that promotes macrophage activation and cytotoxic T lymphocyte function, facilitating clearance of intracellular pathogens. Gene expression profiling of CRSsNP tissues reveals upregulation of IFN-γ signaling pathways and antiviral immunity genes, implicating T cells (Th1 and CD8+), natural killer (NK) cells, and antigen-presenting cells as key players in this inflammatory milieu (25). The sinonasal epithelium, while traditionally viewed as a passive barrier, actively participates in innate immunity through pattern-recognition receptors and secretion of chemokines that recruit and activate these immune cells. Moreover, epithelial dysfunction induced by type 1 cytokines can exacerbate inflammation and tissue remodeling (26). Immune cell composition in type I inflammation includes increased infiltration of Th1 cells, cytotoxic CD8+ T cells, and macrophages, which produce pro-inflammatory mediators such as tumor necrosis factor-alpha (TNF-α) and IFN-γ, perpetuating a non-eosinophilic inflammatory state typical of CRSsNP (25). Viral infections, particularly rhinovirus, can further modulate this immune response; silent rhinovirus infection in nasal tissues is associated with elevated type 1 interferons and IFN-γ expression in healthy controls, although this response is altered in CRS patients (27). The epithelial barrier disruption in CRS may also be influenced by hypoxia-induced factors such as HIF-1α, which downregulates tight junction proteins and promotes inflammation, potentially affecting type I inflammatory pathways (28). Collectively, type I inflammation in CRS represents a complex interplay between innate and adaptive immune responses aimed at microbial clearance but may also contribute to chronic mucosal inflammation and remodeling in CRSsNP. Understanding the cellular and molecular mechanisms underlying this endotype is critical for developing targeted therapies, especially since type 1 immune responses differ fundamentally from type 2 and type 3 pathways that dominate other CRS phenotypes. Mechanistically, this endotype highlights how epithelial pathogen sensing and barrier impairment can sustain interferon-dominant inflammation with limited alarmin-driven T2 escalation. 

#### 2.1.2 Type 2 inflammation and its immunological characteristics

T2 inflammation in CRSis predominantly driven by the cytokinesIL-4, IL-5, and IL-13, which are hallmark mediators of the T helper 2 (Th2) immune response. This immunological pattern is most typically observed in CRSwNP and is closely associated with allergic diseases, underscoring the overlap between CRS and atopic conditions (29, 30). The T2 inflammatory milieu is characterized by the activation and recruitment of eosinophils, basophils, mast cells, and ILC2s, which collectively contribute to tissue remodeling, mucus hypersecretion, and local edema (31, 32). Elevated levels of IL-4 and IL-13 promote immunoglobulin E (IgE) production by B cells, further amplifying allergic inflammation and sustaining eosinophilic infiltration (30, 33).

Eosinophilic infiltration is a defining feature of T2 inflammation in CRS, particularly in CRSwNP, where eosinophils accumulate in the mucosa and nasal polyps, releasing cytotoxic granule proteins such as eosinophil cationic protein (ECP) that contribute to tissue damage and remodeling (24, 29). The presence of eosinophil extracellular traps (EETosis) and Charcot-Leyden crystals further exacerbates local inflammation and epithelial barrier dysfunction (29, 34). Notably, epithelial cells in the sinonasal mucosa not only serve as a physical barrier but also actively participate in T2 inflammation by producing alarmins such as IL-25, IL-33, and TSLP, which activate ILC2s and Th2 cells, perpetuating the inflammatory cascade (35, 36).

Recent advances have highlighted the heterogeneity within T2 inflammation, distinguishing between T2-high and T2-low profiles based on cytokine levels and eosinophil counts. T2-high CRSwNP patients exhibit elevated IL-5, IL-4, and IL-13 levels alongside marked eosinophilia and more severe clinical phenotypes, including increased polyp burden and frequent need for systemic corticosteroids (37). Conversely, T2-low endotypes display lower cytokine expression and reduced eosinophilic inflammation, suggesting variable therapeutic responsiveness (37). The role of novel biomarkers such as cystatin SN (CST1), which is overexpressed in epithelial cells and promotes T2 inflammation, has been implicated in disease pathogenesis and may serve as potential therapeutic targets (38). Among epithelial-derived candidates for noninvasive endotyping, CST1 has emerged as a promising marker linked to T2-high CRS. A recent study integrating single-cell RNA sequencing and protein validation demonstrated that CST1 is selectively expressed in epithelial cells in T2 CRS but not non-T2 CRS, and that cystatin SN is readily detectable in epithelial lining fluid (ELF), where its levels are significantly higher in T2 CRS than in non-T2 CRS and controls. Importantly, ELF cystatin SN correlates with established measures of disease burden (Lund–Mackay CT score, SNOT-22, and JESREC score), inversely correlates with olfactory function, and tracks with tissue/blood eosinophil counts and tissue T2 inflammatory mediators, supporting CST1 as a clinically feasible, noninvasive biomarker for T2 endotyping (39).

Allergic fungal rhinosinusitis (AFRS) is considered a prototypical type 2 endotype of CRSwNP. Patients with AFRS demonstrate elevated serum IgE levels and eosinophilic mucin with abundant eosinophils and IgE−mediated sensitization to fungal antigens. Airway colonization by fungi triggers robust T2 cytokine production (IL−4, IL−5 and IL−13), leading to eosinophilia and IgE elevation. Persistent fungal exposure, however, may also induce neutrophilic inflammation through IL−1, IL−6 and IL−17 pathways. Thus, AFRS exhibits mixed inflammatory signals, but its dominant immunopathologic mechanism is type 2 inflammation. We therefore discuss AFRS within the T2 endotype while noting the potential contribution of Th17−mediated responses in some cases.

Therapeutically, targeting the IL-4/IL-13 axis with biologics such as dupilumab has demonstrated efficacy in reducing polyp size, improving symptoms, and modulating T2 inflammation in CRSwNP patients (40, 41). The downregulation of prostacyclin receptor (IP) expression in CRS mucosa inversely correlates with IL-4, IL-5, and IL-13 levels, indicating its potential as a modulator of T2 inflammation (42). Moreover, environmental factors such as particulate matter (PM_2.5_) can exacerbate T2 inflammation by inducing epithelial cytokines and ILC2 activation, while agents like retinoic acid may mitigate these effects by promoting regulatory innate lymphoid cells (43).

In summary, type 2 inflammation in CRS is a complex immunological process orchestrated by IL-4, IL-5, and IL-13-driven Th2 responses, characterized by eosinophilic infiltration and epithelial-immune cell interactions. This endotype is predominant in CRSwNP and allergic diseases and underlies the pathogenesis, clinical severity, and therapeutic responsiveness of these conditions. Understanding the nuances of T2 inflammation, including its cellular and molecular mediators, is critical for the development of targeted therapies and personalized management strategies in CRS. Thus, T2-high CRSwNP can be conceptualized as an alarmin-initiated and ILC2/Th2-amplified circuit that drives eosinophilic inflammation and remodeling.

#### 2.1.3 Type 3 inflammation and its immunological characteristics

Type 3 inflammation in CRS emerges when epithelial-immune crosstalk preferentially supports Th17 programs, often in the presence of persistent microbial stimulation, dysbiosis, or neutrophil-dominant inflammation. In this endotype, Th17-derived IL-17A primarily induces epithelial and stromal production of neutrophil-attracting chemokines, particularly CXCL8/IL-8, thereby driving neutrophilic recruitment and activation. In contrast, IL-22 acts predominantly on epithelial cells, where it regulates antimicrobial peptide production, epithelial repair, and barrier integrity. Thus, IL-17A and IL-22 represent functionally distinct but complementary components of the Th17-associated mucosal response in CRS. This cytokine milieu is particularly associated with CRS without nasal polyps (CRSsNP), where type 3 inflammation contributes to disease pathogenesis and contributes to disease pathogenesis through mechanisms distinct from eosinophilic, type 2-driven inflammation (25, 26).

The association of type 3 inflammation with bacterial and fungal infections is well documented. IL-17 and IL-22 promote the expression of antimicrobial peptides and enhance epithelial barrier function, which are critical defenses against pathogens. However, in CRS, persistent microbial colonization or infection may drive chronic activation of this pathway, leading to sustained neutrophilic inflammation and tissue damage. Although fungal colonization can induce Th17−mediated responses, AFRS is primarily driven by type 2 inflammation and is discussed in Section 2.1.2 (25, 44).

Neutrophils, as the primary effector cells in type 3 inflammation, play a pivotal role in the chronic inflammatory milieu of CRS. Their recruitment is driven by IL-17-induced chemokines such as CXCL8 (IL-8), which is elevated in CRSsNP patients. Neutrophils contribute to inflammation through the release of proteolytic enzymes, reactive oxygen species, and neutrophil extracellular traps (NETs), which can exacerbate mucosal damage and perpetuate the inflammatory cycle. Calprotectin, a neutrophil-derived protein, has emerged as a biomarker of non-type 2 inflammation in CRS, correlating with neutrophil counts and disease severity, especially in patients with multiple prior surgeries, highlighting the clinical relevance of neutrophilic inflammation (45).

The heterogeneity of CRS is further underscored by geographic and age-related variations in inflammatory endotypes. Studies have shown that type 3 cytokines tend to be more prominent in CRSsNP and non-ECRS subtypes, with an age-associated decline in type 3 mediators observed in some populations, suggesting dynamic immune regulation over time (46). Moreover, the presence of bacterial superantigens may amplify Th17 responses and neutrophilic inflammation, contributing to disease chronicity and severity (47).

Beyond their role in host defense, Th17 cells contribute to chronic auto−immune disorders. Th17 effector cytokines (IL−17A/F, IL−21 and IL−22) drive persistent inflammation in diseases such as rheumatoid arthritis, psoriasis and Crohn’s disease. IL−17 promotes neutrophil recruitment, IL−21 regulates B−cell activation and auto−antibody production, and IL−22 participates in epithelial remodeling. Dysregulated Th17 responses in CRS may similarly lead to tissue remodeling, steroid resistance and refractory disease. Crosstalk between Th17− and Th2−driven pathways can give rise to mixed T2/T3 endotypes, emphasizing the need to consider Th17 biology in both eosinophilic and non−ECRSwNP. This broader perspective situates CRS within the evolving understanding of Th17−mediated pathology.

In summary, type 3 inflammation in CRS is characterized by Th17 cell-mediated production of IL-17 and IL-22, driving neutrophilic infiltration and antimicrobial responses predominantly in CRSsNP. This endotype is closely linked to microbial factors, including bacterial and fungal infections, and involves neutrophil-mediated tissue damage that sustains chronic inflammation. Understanding the immunological features of type 3 inflammation provides insight into the pathophysiology of CRS and identifies potential biomarkers and therapeutic targets distinct from type 2-driven disease processes (25, 26, 45). Importantly, Th17-driven inflammation may coexist with epithelial alarmin signaling, contributing to mixed endotypes and steroid-refractory disease in selected CRS subsets.

#### 2.1.4 Non−eosinophilic CRSwNP: mechanisms of polyp formation beyond type 2 inflammation

Non−eosinophilic CRSwNP represents a clinically important subgroup in which nasal polyps form without prominent eosinophilia. Patients with this phenotype display mixed or non−type 2 immune profiles characterized by neutrophilic inflammation, Th1/Th17 cytokine production and fibrotic remodeling. IFN−γ and IL−17A released by Th1 and Th17 cells disrupt epithelial tight junctions and promote neutrophil chemotaxis, while neutrophils release oncostatin M (OSM) and TGF−β2, which drive mucosal fibrosis and polyp development. Non−eosinophilic CRSwNP tends to show less edema and more fibrosis than eosinophilic CRSwNP and is more prevalent in certain Asian populations. Because these patients often respond poorly to corticosteroids and T2−targeting biologics, management should emphasize microbial control, epithelial barrier repair and modulation of neutrophilic pathways. Recognition of non−eosinophilic CRSwNP is therefore essential for personalized therapy.

### 2.2 Cellular and molecular interactivity in CRS immune endotyping

#### 2.2.1 Epithelial alarmins as upstream immune-programming signals

The sinonasal epithelium is no longer viewed as a passive physical barrier in chronic rhinosinusitis (CRS), but rather as an active immune-regulatory interface that senses environmental insults and initiates downstream inflammatory programs. In response to allergens, pathogens, viral mimics, particulate pollutants, hypoxia, and epithelial injury, nasal epithelial cells release a group of epithelial-derived cytokines, or alarmins, including thymic stromal lymphopoietin (TSLP), interleukin-33 (IL-33), and interleukin-25 (IL-25). These alarmins represent a convergent upstream axis linking epithelial barrier dysfunction to immune endotype polarization, particularly in type 2-high CRS with nasal polyps (CRSwNP) (36, 48–50).

TSLP is primarily produced by respiratory and nasal epithelial cells, but it may also be generated by nasal fibroblasts and hyperplastic basal epithelial cells within nasal polyps. Its expression can be induced by microbial components such as lipopolysaccharide (LPS), viral mimics such as polyinosinic:polycytidylic acid [poly(I:C)], and type 2 cytokines including IL-4 and IL-13 (36). Mechanistically, activation of pattern-recognition receptors, particularly Toll-like receptor 4 (TLR4), triggers downstream MAPK, Akt, and NF-κB signaling pathways, leading to transcriptional upregulation of TSLP in nasal fibroblasts and epithelial cells (51). Hypoxic conditions in inflamed sinonasal tissues further enhance TSLP production through HIF-1α, thereby promoting type 2 immune responses in eosinophilic CRSwNP (ECRSwNP) (22). Genetic evidence also implicates TSLP as a shared susceptibility gene linking CRS with autoimmune and allergic diseases, supporting its central role in mucosal immune regulation (52).

TSLP exerts its immunomodulatory effects through a heterodimeric receptor complex composed of TSLPR and IL-7Rα, which is expressed on group 2 innate lymphoid cells (ILC2s), Th2 cells, and myeloid dendritic cells (53, 54). Through this receptor system, TSLP promotes ILC2 and Th2 activation and induces the production of IL-4, IL-5, and IL-13, thereby amplifying eosinophilic inflammation, mucus hypersecretion, and tissue remodeling in CRSwNP (55). TSLP can also promote basophil activation and cooperate with oncostatin M (OSM) and IL-4 to further enhance epithelial TSLP synthesis, suggesting the existence of a positive feedback loop between epithelial remodeling and type 2 inflammation (56, 57). Clinically, elevated TSLP expression has been detected in nasal polyp tissues and inferior turbinate mucosa of patients with CRSwNP, and is associated with type 2 inflammatory markers, fractional exhaled nitric oxide (FeNO), and SNOT-22 scores (58). Microbiota-derived short-chain fatty acids (SCFAs) may suppress TSLP expression, suggesting that microbial metabolites may modulate epithelial barrier integrity and inflammatory tone (59). Therapeutically, monoclonal antibodies targeting TSLP or its receptor, including tezepelumab and ASP7266, have shown potential in reducing type 2 inflammation in CRSwNP and related airway diseases by inhibiting upstream TSLP signaling and its downstream effects on ILC2s and Th2 cells (60, 61). Tezepelumab has also demonstrated clinical benefit in severe asthma with comorbid CRSwNP, underscoring the translational relevance of TSLP blockade (62, 63).

IL-33 is another pivotal epithelial-derived alarmin in CRS, particularly in eosinophilic CRSwNP. Unlike secreted cytokines, IL-33 is constitutively expressed in the nuclei of epithelial cells, endothelial cells, and fibroblasts, functioning as a sentinel molecule released upon cellular damage or stress. Its expression is regulated at both transcriptional and post-translational levels, and HIF-1α has been identified as an upstream regulator that enhances IL-33 production under hypoxic conditions within inflamed sinonasal tissues (22). IL-33 binds to its receptor ST2, also known as IL1RL1, which is expressed on ILC2s, mast cells, Th2 cells, eosinophils, and regulatory T cells. This interaction activates MyD88-dependent signaling and amplifies type 2 immune responses (64). In CRSwNP, elevated IL-33 expression correlates with eosinophilic infiltration and disease severity. Serum IL-33 and soluble ST2 levels are increased in eosinophilic CRSwNP compared with non-eosinophilic disease and healthy controls, suggesting their potential utility as biomarkers for endotyping and postoperative recurrence prediction (65). MicroRNA-21-5p may further exacerbate mucosal type 2 inflammation by upregulating IL-33 expression through targeting the glucagon-like peptide-1 receptor, indicating an additional regulatory layer controlling IL-33 availability in the nasal mucosa (66).

Functionally, IL-33 is a potent activator of ILC2s, promoting their expansion, survival, and production of IL-4, IL-5, and IL-13 (67). IL-33 stimulation increases the expression of GATA3 and CRTH2 in ILC2s, both of which are critical for type 2 cytokine production; this pathway can be inhibited by agents such as crocin, which reduces type 2 inflammation in eosinophilic nasal polyps (68). IL-33 also influences mast cell activation, and microRNA-221-3p can modulate IL-33-induced cytokine expression by targeting KIT, highlighting the multifaceted actions of IL-33 on immune-cell subsets within the sinonasal mucosa (69). Elevated IL-33 expression in nasal polyp tissues is associated with increased TSLP and IL-25, epithelial remodeling, and barrier dysfunction (50, 70). Environmental factors, including airborne particulate matter such as PM10, can induce IL-33 expression in nasal polyp-derived fibroblasts and epithelial cells, thereby enhancing type 2 inflammation and disease severity (71, 72). Although direct clinical evidence in CRSwNP remains emerging, anti-IL-33 or anti-ST2 strategies have shown therapeutic promise in related airway diseases, suggesting potential relevance for CRS treatment (73).

IL-25, also known as IL-17E, further contributes to the initiation and amplification of type 2 immunity in CRSwNP. It is mainly secreted by nasal epithelial cells in response to epithelial stress, viral infection, and hypoxia. HIF-1α expression positively correlates with IL-25 expression in ECRSwNP, suggesting that hypoxia may promote IL-25 production through HIF-1α-mediated mechanisms (22). Respiratory viruses, including influenza A, can directly stimulate human nasal epithelial cells to produce IL-25, linking viral infection to exacerbation of type 2 inflammation in CRSwNP (74). IL-25 acts through the IL-17RB receptor, which is expressed on ILC2s, Th2 cells, and activated B cells. Binding of IL-25 to IL-17RB on ILC2s enhances their activation and proliferation, leading to increased production of IL-5 and IL-13 through STAT3 phosphorylation (75). IL-25 may also promote antibody production by activated B cells, connecting epithelial alarmin signaling with local humoral immunity (76). Increased IL-25 levels and accumulation of IL-17RB-positive immune cells in nasal polyps support the pathological relevance of this pathway (75, 76). Clinically, elevated IL-25 is associated with type 2 cytokine expression, eosinophilic infiltration, disease severity, and recurrence in CRSwNP (74). Agents such as limonin have been shown to inhibit IL-25-induced STAT3 activation and reduce ILC2-derived type 2 cytokine production, suggesting potential therapeutic value in refractory CRSwNP (75).

Taken together, TSLP, IL-33, and IL-25 act as upstream immune-programming signals that translate epithelial injury, hypoxia, microbial stimulation, and environmental exposure into type 2 immune activation. Rather than functioning as isolated mediators, these alarmins form an interconnected epithelial checkpoint that shapes ILC2 activation, Th2 polarization, eosinophilic inflammation, local IgE responses, and tissue remodeling. Their expression levels and downstream signatures, including eosinophilia, local or total IgE, and type 2 cytokine profiles, may therefore provide clinically actionable biomarkers for identifying T2-high disease. Meanwhile, emerging strategies targeting TSLP, IL-33, and IL-25 pathways highlight the therapeutic potential of intervening at an upstream epithelial checkpoint rather than only suppressing downstream effector cytokines.

#### 2.2.2 The alarmin–ILC2/Th2 axis in T2-high CRSwNP

The alarmin–ILC2/Th2 axis represents the central immune circuit underlying T2-high CRSwNP. ILC2s are innate immune cells characterized by the absence of lineage markers for T, B, and myeloid cells and by the expression of CD127, CRTH2, and ST2. They rapidly respond to epithelial-derived alarmins, particularly IL-25, IL-33, and TSLP, without requiring antigen-specific stimulation. Engagement of these alarmins with their corresponding receptors activates intracellular pathways such as STAT5, STAT6, NF-κB, AP-1, and STAT3, resulting in robust production of type 2 cytokines, including IL-4, IL-5, and IL-13 (77, 78).

In CRSwNP, ILC2s accumulate within nasal polyp tissues and serve as major early sources of type 2 cytokines. ILC2-derived IL-5 promotes eosinophil recruitment, activation, and survival, whereas IL-13 contributes to goblet cell metaplasia, mucus hypersecretion, and epithelial barrier dysfunction. ILC2s may also resist regulatory T-cell-mediated suppression, further sustaining local type 2 inflammation. Their clinical relevance is supported by evidence that biologic therapies targeting IL-4/IL-13 signaling, such as dupilumab, can modulate circulating ILC2 proportions and function, linking ILC2 activity to disease burden and therapeutic response (79, 80). In addition, a TLR4-positive ILC2 subset with features of trained immunity has been identified in CRSwNP, suggesting that non-specific immune memory may help maintain persistent type 2 responses in refractory disease (81, 82).

ILC2s also exhibit plasticity and metabolic heterogeneity. CD45RO-positive inflammatory ILC2s have been identified in inflamed mucosal tissues and are associated with steroid resistance and metabolic reprogramming, potentially contributing to persistent inflammation in CRSwNP (77, 78). Beyond cytokine-mediated activation, lipid mediators and metabolic pathways further regulate ILC2 function. Dysregulated fatty acid metabolism and reduced 12/15-lipoxygenase-derived lipid mediators can augment IL-33-induced ILC2 proliferation and cytokine production, whereas specialized pro-resolving mediators such as maresin 1 and resolvin D1 can suppress ILC2 cytokine secretion. Environmental factors such as PM_2_._5_ may exacerbate type 2 inflammation by enhancing epithelial cytokine production and expanding ILC2 populations, while retinoic acid may induce regulatory ILC phenotypes and IL-10 production, thereby mitigating inflammation (83). These findings indicate that ILC2 responses are regulated not only by epithelial alarmins but also by lipid metabolism, environmental exposure, and local tissue context.

Th2 cells provide the adaptive immune counterpart to the ILC2 response and are essential for sustaining type 2 inflammation in CRSwNP. Th2 differentiation is driven by antigen stimulation in an IL-4-rich milieu and depends on transcription factors such as GATA3. Compared with non-polypoid CRS or healthy mucosa, nasal polyp tissues show increased Th2 cell infiltration and enhanced Th2 polarization (84). Single-cell transcriptomic analyses have identified distinct Th2 subsets in nasal polyps, including CD109-positive CRTH2-negative Th2 cells that produce IL-10, indicating heterogeneity within the Th2 compartment (84). Additional regulators such as Schnurri 3 (SHN3), which promotes IL-4 production and late-phase Th2 polarization, and Malat1, which modulates cytokine expression in female Th2 cells, suggest that both transcriptional and host-related factors influence Th2 behavior in inflammatory disease (85, 86).

The pathogenic effects of Th2 cells are mediated primarily through IL-4, IL-5, and IL-13. IL-4 promotes Th2 differentiation and B-cell class switching to IgE, contributing to local IgE elevation in nasal polyps (21). IL-5 is essential for eosinophil recruitment, activation, and survival, thereby driving eosinophilic infiltration in CRSwNP (21, 87). IL-13 induces mucus hypersecretion, goblet cell hyperplasia, and epithelial barrier dysfunction, further compromising mucosal integrity (88, 89). These cytokines act synergistically with ILC2-derived mediators to reinforce epithelial remodeling and chronic inflammation (79). Extracellular vesicles released by Th2 cells may also carry cytokines such as IL-3, which promote eosinophil survival and prolong airway eosinophilia, suggesting an additional mechanism of Th2-mediated inflammation (90).

The Th2–eosinophil interaction is a key effector axis in T2-high CRSwNP. Th2-derived IL-5 drives eosinophil recruitment and activation, whereas eosinophils release cytotoxic granule proteins and inflammatory mediators that perpetuate epithelial injury, tissue remodeling, and polyp formation (91). This crosstalk is modulated by dendritic cells, which provide costimulatory signals that enhance Th2 responses (92), and by chemokine networks shaped by Th2 cells and macrophage subsets, which facilitate the recruitment of eosinophils and other inflammatory cells (93, 94). Periostin, induced by Th2 cytokines, further contributes to extracellular matrix remodeling and osteitis in ECRS (95, 96). Together, these mechanisms explain why T2-high CRSwNP often presents with eosinophilia, mucus hypersecretion, edema, polyp burden, and responsiveness to biologics targeting IL-4Rα or IL-5 pathways.

Thus, the alarmin–ILC2/Th2 axis can be conceptualized as an epithelial-initiated and immune-cell-amplified circuit. Epithelial alarmins activate ILC2s and Th2 cells; ILC2s rapidly provide innate type 2 cytokines; Th2 cells sustain adaptive type 2 polarization; eosinophils and B cells execute tissue-damaging and humoral effector functions; and epithelial remodeling feeds back to sustain alarmin production. This interactive circuit underlies the pathobiology and therapeutic responsiveness of T2-high CRSwNP.

#### 2.2.3 The Th17–neutrophil axis in type 3 and mixed endotypes

In contrast to T2-high CRSwNP, type 3 inflammation is characterized by Th17-associated cytokines and neutrophil-dominant inflammation. Th17 cells are a distinct subset of CD4+ T helper cells whose differentiation is driven by cytokines such as TGF-β, IL-6, IL-1β, and IL-23 through transcription factors including RORγt and STAT3. They produce IL-17A and IL-22, which have related but functionally distinct roles in mucosal immunity. IL-17A primarily induces epithelial and stromal production of neutrophil-attracting chemokines, especially CXCL8/IL-8, thereby promoting neutrophilic recruitment and activation. In contrast, IL-22 acts mainly on epithelial cells, where it regulates antimicrobial peptide production, epithelial repair, and barrier integrity. Therefore, IL-17A and IL-22 should be viewed as complementary but non-identical components of the Th17-associated mucosal response in CRS.

Neutrophilic inflammation is particularly relevant in CRSsNP, non-eosinophilic CRSwNP, and mixed T2/T3 endotypes. Neutrophils contribute to persistent inflammation not only as effector cells but also as active regulators of adaptive immunity. Neutrophil extracellular traps (NETs), which are web-like DNA structures released upon neutrophil activation, can directly promote Th17 differentiation through TLR2-mediated STAT3 phosphorylation, establishing a positive feedback loop between neutrophils and Th17 cells (97). This interaction is consistent with observations from severe asthma and other airway inflammatory diseases, where Th17-dominant neutrophilic inflammation is often resistant to corticosteroids (98, 99).

The Th17–neutrophil axis is also closely linked to tissue remodeling and fibrosis. IL-17A can stimulate epithelial cells and fibroblasts to produce pro-fibrotic factors and extracellular matrix components, contributing to airway remodeling. In CRS, particularly in CRSsNP and non-type 2 disease, such remodeling may manifest as mucosal thickening, fibrosis, and loss of normal tissue architecture. Neutrophil-derived signals, including those mediated by CARD9, can potentiate Th17 responses and thereby promote fibrosis and chronic inflammation (100). Cellular stress pathways may further reinforce this axis. For example, the unfolded protein response sensor IRE1, activated by cytokines such as IL-23, enhances Th17 secretory function and neutrophilic infiltration, linking cellular stress responses to inflammation and fibrosis (101, 102). Therapeutic modulation of this axis, such as vitamin D3 priming of dendritic cells or targeting the BATF transcription factor, has shown potential in attenuating Th17-driven neutrophilic inflammation and tissue damage (99, 103).

Within CRS tissues, neutrophils maintain inflammation through cytokine release, NET formation, and reciprocal interaction with Th17 cells. IL-17A promotes neutrophil recruitment and activation, whereas neutrophils enhance Th17 differentiation and survival through NETs and other mediators (104, 105). Additional molecular signals, including IL-6, IL-1β, and granulocyte colony-stimulating factor (G-CSF), regulate neutrophil survival and apoptosis, influencing the degree of neutrophilic inflammation in CRS (106, 107). These interactions may help explain why neutrophil-predominant CRS is often refractory to corticosteroids and less responsive to therapies designed for T2-high disease.

Importantly, type 3 inflammation should not be interpreted as entirely separate from type 2 inflammation. Mixed T2/T3 endotypes may occur when epithelial alarmin signaling coexists with microbial stimulation, dysbiosis, or persistent neutrophilic inflammation. In such settings, eosinophilic inflammation, Th17 activation, neutrophil recruitment, NET formation, and epithelial remodeling may coexist within the same tissue microenvironment. Recognition of this overlap is critical for clinical endotyping, because patients with mixed or non-T2 inflammatory patterns may require strategies beyond conventional corticosteroids or T2-targeting biologics, including microbial control, barrier restoration, and therapies targeting IL-17-related or neutrophil-associated pathways.

#### 2.2.4 Regulatory and humoral immune modules: Tregs, B cells, and plasma cells

Although effector type 2 and type 3 pathways drive much of the inflammatory burden in CRS, immune regulation and humoral immunity also shape disease persistence, recurrence, and therapeutic heterogeneity. Regulatory T cells (Tregs), characterized by CD4, CD25, and Foxp3 expression, maintain immune homeostasis by suppressing excessive or aberrant immune responses. In CRSwNP, impaired Treg function or reduced Treg abundance contributes to local immune imbalance. CRSwNP tissues show decreased frequencies of CD4+CD25+Foxp3+ Tregs and reduced expression of immunosuppressive cytokines such as TGF-β and IL-10, both of which are critical for Treg-mediated suppression (108). This regulatory deficiency is associated with heightened type 2 and type 3 inflammation, eosinophilic infiltration, and increased eosinophil cationic protein levels.

Tregs in CRSwNP also display phenotypic alterations. Reduced expression of neuropilin-1 and Helios, markers associated with Treg stability and suppressive function, suggests impaired regulatory capacity within nasal polyp tissues (24). Experimental evidence supports the therapeutic potential of Treg restoration: adoptive transfer of CD4+CD25+Foxp3+ Tregs into ECRS mice attenuates inflammatory cytokine and chemokine production and reduces eosinophil recruitment without disturbing systemic immune balance (24). In addition to CD4+ Tregs, CD8+CD25+Foxp3+ Tregs may regulate neutrophilic inflammation in neutrophilic CRSwNP. Although these cells are reduced in nasal polyps, *in vitro* administration suppresses pro-inflammatory mediators such as myeloperoxidase, IFN-γ, IL-1β, and TNF-α; however, local budesonide treatment does not significantly enhance their frequency or function, suggesting that current therapies may be insufficient to restore this regulatory subset (109).

Treg function is further shaped by interactions with immune and stromal components. The TNF–TNFR2 axis, preferentially expressed on Tregs, is important for Treg activation and expansion and may serve as a negative feedback mechanism controlling chronic inflammation in CRSwNP. Stromal-derived laminin α5 may also influence Treg migration and suppressive function in mucosal tissues (110). Notably, Tregs in CRS may exhibit functional plasticity. A subset of Foxp3+ Tregs in nasal polyps can produce IL-17A, a cytokine typically associated with Th17 responses, suggesting that under inflammatory conditions Tregs may acquire pro-inflammatory features and participate in polyp pathogenesis (111). Thus, CRS is not only characterized by exaggerated effector inflammation but also by failure or reprogramming of local immune regulation.

Local humoral immunity represents another important layer of CRS immune regulation. Within inflamed sinonasal tissues, B cells undergo clonal expansion and differentiate into plasma cells that produce immunoglobulins, including IgE, IgG, and IgA. In CRSwNP, increased B-cell and plasma-cell infiltration correlates with elevated local IgE, which can amplify mast-cell and eosinophil activation and reinforce type 2 inflammation (112, 113). The marginal-zone B-cell marker MZB1 is highly expressed in plasma cells and mature B cells and is associated with enhanced local IgE synthesis, suggesting a role in sustaining humoral inflammation in T2-high CRSwNP (112). B-cell activation and plasma-cell homing are also regulated by transcriptional programs such as Krüppel-like factor 2 (KLF2), and dysregulation of these processes may contribute to pathological antibody production and chronic inflammation (114, 115).

B-cell and plasma-cell responses may also contribute to mixed endotypes. Although local humoral immunity is most prominent in T2-high CRSwNP, where IL-4 and IL-13 support IgE class switching, Th17-associated cytokines such as IL-21 and IL-17-related signaling may also promote B-cell activation and local antibody responses in chronic mucosal inflammation. This suggests that local immunoglobulin compartments, including IgE, IgA, and IgG, and plasma-cell signatures may complement cytokine and cellular markers when defining endotypes and predicting therapeutic responses. In CRS, the CD40/CD40L axis is critical for B-cell maturation and antibody production, and altered soluble CD40 ligand levels correlate with disease severity, indicating dysregulated peripheral and local B-cell function (116). In aspirin-exacerbated respiratory disease, nasal polyp antibody-secreting cells show increased proliferation and IL-5 receptor α expression, potentially promoting plasma-cell expansion and IgE production (117). *Staphylococcus aureus* can also stimulate IgE production through memory B cells in nasal polyps, demonstrating how microbial factors may directly influence local humoral immunity in CRS (118).

Beyond antibody production, regulatory B cells and innate-like B-cell subsets may contribute to immune homeostasis, although CRS-specific evidence remains limited. Bregs normally suppress inflammation, but their alteration in chronic inflammatory conditions may contribute to disease chronicity (119). Interactions among B cells, T cells, and stromal cells may form feedback loops that sustain inflammation and tissue remodeling (120). Innate-like B-cell subsets, such as B-1a cells, can modulate inflammation through cytokine production and natural antibody secretion, but their localization and functional contribution in CRS remain largely indirect and hypothesis-generating (121). Future tissue-resolved phenotyping and functional studies are needed to determine whether these subsets influence endotype stability, recurrence risk, or treatment response.

Collectively, regulatory and humoral immune modules contribute to CRS by shaping the balance between immune activation and immune tolerance. Treg deficiency or plasticity may permit uncontrolled type 2 or type 3 inflammation, whereas B-cell and plasma-cell activation may sustain local IgE production and chronic mucosal inflammation. Therapeutic strategies aimed at restoring regulatory circuits, modulating the TNF–TNFR2 axis, targeting B-cell activation pathways, or interfering with cytokines such as IL-5 and IL-21 that support plasma-cell responses may offer additional approaches for difficult-to-treat CRS (122, 123).

#### 2.2.5 Integrated view: from epithelial sensing to endotype-specific therapy

The cellular and molecular interactions described above support a unified model of CRS immune endotyping. In T2-high CRSwNP, epithelial injury, hypoxia, allergens, viruses, and pollutants induce TSLP, IL-33, and IL-25 release. These alarmins activate ILC2s and Th2 cells, which produce IL-4, IL-5, and IL-13, leading to eosinophil recruitment, local IgE production, mucus hypersecretion, epithelial remodeling, and polyp growth. This circuit provides the mechanistic basis for therapies targeting IL-4Rα, IL-5, IgE, and upstream epithelial alarmins.

In type 3 or mixed endotypes, persistent microbial stimulation, dysbiosis, epithelial stress, and neutrophilic inflammation promote Th17 activation, IL-17A-driven CXCL8-mediated neutrophil recruitment, NET formation, and tissue remodeling. IL-22 may support epithelial antimicrobial defense and barrier repair, but persistent or dysregulated type 3 activation can contribute to chronic inflammation, fibrosis, and steroid resistance. In parallel, impaired Treg function and dysregulated B-cell/plasma-cell responses further destabilize local immune homeostasis and contribute to endotype overlap.

This integrated epithelial–immune framework explains why CRS cannot be adequately classified solely by the presence or absence of nasal polyps. Instead, CRS should be understood as a spectrum of immune-inflammatory states shaped by epithelial sensing, alarmin release, innate and adaptive immune-cell crosstalk, regulatory failure, humoral amplification, and tissue remodeling. Recognizing these interactions provides a mechanistic foundation for biomarker-based endotyping and personalized therapy selection.

### 2.3 Differences in CRS immune responses among different populations

#### 2.3.1 Differences in immune endotypes between Asian and Western patients with CRS

Having outlined epithelial sensing mechanisms and downstream immune endotype programming, we next discuss how population heterogeneity and genetic/environmental factors shape the prevalence and clinical expression of these pathways.

CRSexhibits notable immunological heterogeneity that varies significantly between Asian and Western populations, influencing both clinical manifestations and therapeutic approaches. In Asian patients, CRS often presents as a mixed inflammatory profile characterized by both type 1 (Th1) and type 3 (Th17) immune responses. This mixed endotype contrasts with the predominantly type 2 (Th2) inflammation observed in Western patients with CRS with nasal polyps (CRSwNP). The type 2 immune response is typically marked by eosinophilic infiltration, elevated levels of interleukins such as IL-4, IL-5, and IL-13, and is strongly associated with allergic inflammation and eosinophilic mucus production. Eosinophilic inflammation, which is a hallmark of type 2 immunity, is linked to mucus hyperconcentration and increased viscosity, contributing to persistent sinonasal obstruction and tissue remodeling in CRS patients (124). This eosinophilic dominance is more frequently reported in Western CRSwNP, where it correlates with more severe disease phenotypes and a higher likelihood of comorbid asthma.

In contrast, Asian CRS patients often exhibit a neutrophil-predominant inflammatory pattern, indicative of type 1 and type 3 immune responses. These immune pathways involve IFN-γ and IL-17 cytokines, which mediate neutrophilic inflammation and are important for host defense against bacterial infections. The neutrophilic inflammation seen in Asian CRS may be less responsive to corticosteroids, which are more effective in suppressing type 2 eosinophilic inflammation. This difference in immune endotypes is clinically relevant, as it explains the variability in treatment responses between populations. For example, corticosteroid therapy, including systemic and topical formulations such as budesonide, has demonstrated efficacy primarily in type 2-dominant CRS by reducing eosinophilic infiltration and sinonasal inflammation (125). However, in Asian patients with mixed or neutrophilic inflammation, corticosteroids may be less effective, necessitating alternative or adjunctive therapies targeting neutrophilic pathways or bacterial colonization.

The immunological divergence also impacts the clinical presentation of CRS. Western patients with type 2 inflammation often present with nasal polyposis, severe eosinophilic mucin, and comorbid allergic diseases such as asthma. Conversely, Asian patients may show less prominent nasal polyposis and more chronic mucosal thickening with neutrophilic infiltrates, reflecting a different pathophysiological mechanism. These differences underscore the need for tailored diagnostic and therapeutic strategies based on immune profiling. Biomarkers such as eosinophil counts, cytokine profiles, and mucosal histopathology can guide personalized treatment, improving outcomes by aligning therapy with the underlying immune endotype.

Furthermore, the immune endotype differences have implications for emerging biologic therapies targeting type 2 inflammation, such as anti-IL-5 or anti-IL-4 receptor antibodies. These agents have shown promise in Western CRSwNP patients but may have limited efficacy in Asian patients with mixed or non-type 2 inflammation. Understanding these immunological distinctions is crucial for optimizing clinical management and developing region-specific treatment guidelines. In summary, the immune endotype disparities between Asian and Western CRS patients reflect fundamental variations in disease pathogenesis, clinical features, and therapeutic responsiveness, highlighting the importance of immune phenotyping in guiding effective clinical care (124, 125).

#### 2.3.2 Regulation of immune responses by genetic and environmental factors

The regulation of immune responses in CRSis profoundly influenced by a complex interplay between genetic susceptibility and environmental exposures, which together shape the disease phenotype and progression. Genetic predisposition affects immune cell function by modulating key pathways involved in inflammation, epithelial barrier integrity, and immune regulation. Genome-wide association studies and candidate gene analyses have identified numerous genetic variants associated with CRS, particularly CRSwNP. For example, polymorphisms in genes related to innate and adaptive immunity, such as those encoding major histocompatibility complex (MHC) molecules, cytokines like TNF-α, and olfactory receptors (e.g., OR2A25), have been implicated in altering immune responses and increasing disease risk (126, 127). The missense variant rs61731397-C in OR2A25, predominantly expressed in T cells and nasal epithelial cells, has been shown to reduce protein stability and expression, potentially impairing mucosal immune defense and contributing to CRS pathogenesis (127). Moreover, genetic polymorphisms in cytokine genes, including those involved in Th2 and Th17 pathways (e.g., IL-4, IL-13, IL-17), influence susceptibility and severity of allergic fungal rhinosinusitis, highlighting the role of host genetics in shaping immune polarization (128).

Environmental exposures and lifestyle factors further modulate immune responses through direct effects on epithelial barrier function and by shaping the local microbiome. Airborne allergens, fungal species such as Alternaria alternata, and bacterial colonization (e.g., *Staphylococcus aureus*) contribute to persistent mucosal inflammation by activating innate immune receptors and promoting type 2 immune responses characterized by eosinophilic infiltration and IgE production (129, 130). The epithelial barrier, serving as the first line of defense, is susceptible to disruption by environmental insults including pollutants and microbial products, leading to increased permeability and dysregulated immune activation (131). Oxidative stress induced by environmental factors exacerbates this barrier dysfunction and perpetuates chronic inflammation in CRS (132). Lifestyle factors such as smoking and exposure to pollutants have been associated with altered immune cell profiles and worsened clinical outcomes, although detailed mechanistic studies remain limited.

A critical component of immune regulation involves pattern recognition receptors such as toll-like receptors (TLRs) and nucleotide-binding oligomerization domain (NOD) receptors, which detect microbial components and initiate immune signaling cascades. Differential expression and functional variations in these receptors have been observed in CRS patients, influencing the magnitude and quality of immune responses. For instance, increased expression of TLRs in nasal epithelial cells upon fungal exposure can amplify Th2-skewed inflammation and eosinophil activation, contributing to nasal polyp formation (129). Genetic variations affecting TLR and NOD receptor pathways may alter host susceptibility to microbial colonization and immune dysregulation, although comprehensive data in CRS are still emerging. Additionally, bitter taste receptors such as TAS2R38, expressed in sinonasal epithelial cilia, have been linked to innate immune defense against bacterial pathogens. Genotypic differences in TAS2R38 influence bacterial colonization patterns and immune responses, although their impact on surgical outcomes in CRS remains inconclusive (133).

Epigenetic modifications represent another layer through which environmental factors regulate gene expression and immune function in CRS. DNA methylation changes in genes like TSLP, IL-8, and others involved in inflammation and barrier function have been documented, suggesting that environmental exposures can induce heritable but reversible alterations in immune regulation (134). MicroRNAs (miRNAs) also modulate immune pathways and tissue remodeling, linking environmental stimuli to gene expression changes that influence CRS pathogenesis.

In summary, the regulation of immune responses in CRS is orchestrated by a multifaceted network of genetic susceptibilities, environmental exposures, and receptor-mediated signaling pathways. Genetic variants influence immune cell functions and epithelial integrity, while environmental factors such as allergens, microbes, and pollutants modulate immune activation and barrier status. Differential expression and function of immune receptors like TLRs and NODs mediate host-microbe interactions and inflammatory responses. Understanding these complex interactions is essential for developing personalized therapeutic strategies targeting the underlying immunopathology of CRS. Continued research integrating genomics, epigenetics, and environmental assessments will be critical to unraveling the precise mechanisms governing immune regulation in CRS and improving clinical outcomes.

### 2.4 Clinical significance and future directions of CRS immune-inflammatory typing

#### 2.4.1 Endotype-guided precision therapy in CRS

Immune-inflammatory endotyping provides a mechanistic framework for translating CRS heterogeneity into individualized treatment strategies. Instead of relying solely on conventional clinical phenotypes, such as CRSwNP and CRSsNP, endotyping stratifies patients according to the dominant inflammatory pathways that drive disease persistence, recurrence, and therapeutic responsiveness. In this context, T2-high CRS, characterized by elevated IL-4, IL-5, IL-13, eosinophilic inflammation, and local or systemic IgE responses, currently represents the most clinically actionable endotype. Biologic agents targeting IL-5, IL-4 receptor alpha, and IgE have demonstrated efficacy in reducing nasal polyp burden, improving symptom control, and decreasing the need for surgery in patients with T2-dominant CRSwNP (60, 61). These advances mark a shift from broadly anti-inflammatory approaches, such as corticosteroids and antibiotics, toward mechanism-based precision medicine.

Biomarker-based stratification is central to this transition. Blood eosinophils and serum total IgE are practical systemic indicators, but they may incompletely capture local mucosal inflammation. Epithelial lining fluid (ELF) and nasal secretion biomarkers may more directly reflect epithelial–immune crosstalk within the sinonasal microenvironment. Among them, CST1/cystatin SN has emerged as a promising noninvasive marker for T2 CRS. ELF CST1 showed strong performance in identifying T2 CRS, with an AUC of 0.894 and 0.936 in a validation cohort, exceeding the discriminative ability of blood eosinophils, serum total IgE, and JESREC score. At a reported cut-off of 112.5 ng/mg, ELF cystatin SN achieved 75.0% sensitivity and 92.0% specificity, supporting its potential utility for practical T2 endotyping and treatment stratification (39). Other mucus or secretion biomarkers, including IL-5, periostin, and CST-2, may further support patient stratification and longitudinal monitoring of therapeutic responses (62, 63).

However, the clinical translation of immune endotyping remains incomplete. T1 and T3 inflammatory patterns, characterized by IFN-γ-driven, IL-17-associated, or neutrophil-dominant responses, often present with non-eosinophilic or mixed phenotypes and are generally less responsive to corticosteroids and currently available T2-targeting biologics (64). Therapies targeting neutrophilic inflammation, IL-17-related pathways, stromal remodeling, or fibroblast-mediated polypogenesis remain under investigation, and clinically validated options for non-T2 CRS are still limited (65). Therefore, the current clinical value of endotyping is strongest for identifying T2-high disease and guiding biologic selection, whereas non-T2 and mixed endotypes represent major unmet needs for future therapeutic development.

To align this therapeutic framework with current consensus nomenclature, CRS endotypes should be interpreted as partially overlapping and dynamically regulated immune modules rather than rigid disease categories. The EAACI Task Force Report on CRS endotyping provides an important consensus-based framework for integrating T1, T2, and T3 inflammatory pathways into clinical and translational research (135, 136). This perspective is particularly important because many patients exhibit mixed or shifting immune profiles over time, especially after surgery, corticosteroid exposure, biologic treatment, infection, or environmental stimulation.

#### 2.4.2 A Stepwise algorithm for therapy selection and response monitoring

A pragmatic endotype-guided algorithm should integrate clinical phenotype, local and systemic biomarkers, tissue remodeling features, and treatment response. The first step is to identify T2-high disease. Tissue, ELF, or nasal secretion markers reflecting T2 activation, such as IL-5/IL-13 signatures, periostin, eosinophilic mucin, CST1, and increased local IgE, together with systemic indicators such as blood eosinophils and serum total IgE, support a T2-high endotype (39, 62, 63). This step is clinically important because T2-high CRSwNP is currently the endotype most likely to benefit from available biologic therapies.

The second step is to match therapy to the dominant inflammatory axis. Patients with prominent IL-4/IL-13-driven epithelial remodeling, mucus hypersecretion, and barrier dysfunction may preferentially benefit from IL-4Rα blockade, whereas those with marked eosinophilia may be candidates for IL-5 pathway targeting. Patients with strong IgE-associated features may benefit from anti-IgE therapy (60, 61). In patients with broad activation of multiple downstream type 2 cytokines, upstream epithelial alarmin blockade, such as targeting TSLP or IL-33 signaling, may represent a rational strategy to intercept type 2 programming at an earlier stage (22, 36).

The third step is to recognize non-T2 or mixed endotypes. In patients lacking eosinophilic or T2 signatures, evaluation should emphasize neutrophilic inflammation, IL-17-related signals, neutrophil/NET-associated features, microbial burden, and structural remodeling. These patients may respond less consistently to corticosteroids and T2-targeting biologics, and management may need to prioritize microbial control, epithelial barrier repair, and carefully selected immunomodulatory strategies (64, 65).

The fourth step is longitudinal response monitoring. Reassessment of compartment-specific biomarkers, including nasal secretion or tissue cytokines, blood eosinophils, serum IgE, and selected epithelial markers, may support early identification of responders, detection of relapse risk, and rational switching or escalation of therapy. Nevertheless, the clinical implementation of multiplex nasal secretion or ELF biomarkers remains constrained by translational bottlenecks, including non-standardized sampling procedures, inter-platform variability, uncertain universal cut-off values, cost, and limited availability across healthcare systems. Therefore, broad adoption of biomarker-driven algorithms will require multicenter validation and harmonization of assay platforms before integration into global clinical workflows. However, the clinical implementation of multiplex nasal secretion or epithelial lining fluid biomarkers remains constrained by translational bottlenecks, including non-standardized sampling procedures, inter-platform variability, uncertain universal cut-off values, cost, and limited availability across healthcare systems. Therefore, broad adoption of biomarker-driven algorithms will require multicenter validation and harmonization of assay platforms before integration into global clinical workflows.

#### 2.4.3 Epithelial and stromal targets beyond downstream type 2 cytokines

Although currently available biologics mainly target downstream type 2 cytokines or IgE, epithelial-derived cytokines represent upstream therapeutic targets that may intercept inflammatory programming before full activation of the ILC2/Th2/eosinophil axis. TSLP, IL-33, and IL-25 are released by sinonasal epithelial cells in response to allergens, pathogens, pollutants, viral infection, and hypoxia. HIF-1α upregulation under hypoxic conditions enhances the expression of IL-25, IL-33, and TSLP, thereby promoting type 2 inflammation and eosinophilic infiltration (22). This mechanistic link provides the rationale for targeting epithelial alarmins in eosinophilic CRS.

Among epithelial alarmins, TSLP has been most extensively studied. TSLP promotes dendritic-cell activation, Th2 polarization, and downstream type 2 cytokine production, and its blockade has shown promise in preclinical and early clinical contexts. Targeting TSLP may therefore open new therapeutic avenues for CRSwNP, although long-term safety, durability, and patient-selection criteria remain to be established (36). IL-33 and IL-25 also activate ILC2s and promote Th2 cytokine production, making them attractive upstream targets; however, their therapeutic development in CRS remains less mature than that of TSLP.

Upstream intervention may also extend beyond direct cytokine blockade. The protease-activated receptor 2 (PAR-2)/IL-13 receptor α1 pathway has been implicated in epithelial cytokine release, suggesting that PAR-2 antagonism may indirectly reduce alarmin-driven eosinophilic inflammation (66). Similarly, strategies that restore epithelial integrity may complement immune-targeted therapy. The sinonasal epithelium is both a physical barrier and an immune-regulatory interface, and epithelial dysfunction, including impaired mucociliary clearance, tight-junction disruption, and aberrant remodeling, contributes to CRS pathogenesis (67, 68). Therapeutic approaches that combine epithelial barrier restoration with modulation of epithelial-derived cytokines may therefore interrupt the vicious cycle of epithelial damage and chronic inflammation.

Recent single-cell studies suggest that epithelial subsets, including basal cells and tuft cells, participate in the balance between tissue repair and chronic inflammation (69). IL-13 can induce periostin expression predominantly in basal epithelial cells, linking epithelial remodeling to type 2 inflammation (70). Pharmacological agents such as azithromycin may enhance epithelial repair and reduce type 1 inflammation, suggesting that epithelial barrier modulation can have broader immunoregulatory effects (50). Inhibitors of APE1 redox function have also been shown to reduce epithelial-derived IL-25 production and type 2 cytokine levels while improving epithelial barrier function and mitigating oxidative stress (71). In addition, fibroblast- and epithelial cell-derived exosomes may contribute to epithelial–mesenchymal transition and tissue remodeling, offering another potential therapeutic layer (72). Together, these findings support a therapeutic model in which epithelial homeostasis, alarmin blockade, and downstream cytokine inhibition are integrated according to the patient’s dominant endotype.

#### 2.4.4 Immune-cell interactions and multi-omics strategies for future endotyping

Future refinement of CRS endotyping will require deeper understanding of immune-cell interactions and their spatial organization within the sinonasal mucosa. Single-cell RNA sequencing and spatial transcriptomics have begun to reveal complex immune signaling axes in CRS, especially in eosinophilic CRSwNP. Dysregulated CD4+ and CD8+ T-cell subsets, macrophage–eosinophil recruitment, mast-cell enrichment, and altered immune-cell survival programs have been identified, highlighting the complexity of inflammatory crosstalk in the sinonasal microenvironment (73). Pathways such as IL2–STAT5 may promote survival and proliferation of pathogenic immune cells, whereas inhibition of apoptotic pathways may contribute to persistent inflammation. Integrin complexes, including ITGAM/ITGB2 and ICAM-3, are upregulated both systemically and locally in CRSwNP, suggesting roles in immune-cell adhesion, transmigration, and amplification of inflammatory signaling (74). In parallel, cytokine networks such as the IL-33/ST2 axis activate ILC2s, mast cells, eosinophils, and Th2 cells, thereby reinforcing type 2 inflammation (75). Immune checkpoint pathways and neuroimmune signaling have also emerged as regulatory layers that may influence immune-cell activation and inflammatory outcomes (76).

The reciprocal interaction between immune cells and structural cells is equally important. Epithelial cells release IL-25, IL-33, and TSLP to prime type 2 immunity, while infiltrating immune cells can reshape epithelial function and barrier integrity (35). Distinct epithelial populations, including basal progenitor cells and tuft cells, interact dynamically with immune cells and contribute to tissue remodeling and nasal polyp formation (69). In ECRS, eosinophil extracellular traps can stimulate epithelial cells, amplify eosinophilic inflammation, and impair mucociliary function (35, 77). Neutrophils in nasal polyps release OSM, which modulates epithelial and fibroblast responses, illustrating bidirectional communication between immune and structural cells (78). Epithelial barrier dysfunction, characterized by tight-junction disruption, further facilitates immune-cell infiltration and chronic inflammation (79). Environmental factors such as PM_2_._5_ may exacerbate epithelial injury and inflammation, with potential sex-specific immune effects influencing disease progression (80).

Multi-omics technologies provide a powerful platform for translating these cellular interactions into clinically useful endotypes. Single-cell RNA sequencing, spatial transcriptomics, proteomics, metabolomics, and integrated bioinformatics enable high-resolution characterization of cellular heterogeneity, functional states, and intercellular communication networks within the sinonasal microenvironment (81). Multi-omics integration has identified differentially expressed genes and proteins associated with ECRS, including pathways related to angiogenesis, cell motility, and immune responses (82). Single-cell analyses have delineated expanded CD4+ and CD8+ T-cell populations, B cells, tissue-resident macrophages, and monocyte-derived macrophages, highlighting differences between eosinophilic and non-eosinophilic CRS phenotypes (83). Spatial transcriptomics further reveals the spatial organization of immune and epithelial cells, conserved tissue-remodeling features, and receptor–ligand interactions that may sustain chronic inflammation (137). These approaches can also identify novel biomarkers and therapeutic targets, including integrins, cytokine signaling pathways, and metabolic regulators (85). The integration of metabolomics with cytokine profiling may uncover immunometabolic reprogramming that influences inflammatory responses and provides additional opportunities for intervention (86).

Overall, future CRS endotyping should move toward a multidimensional framework that integrates clinical phenotype, tissue and secretion biomarkers, epithelial and immune-cell states, spatial organization, microbiome and environmental exposure, and longitudinal treatment response. Such a framework will support more precise disease classification, improve biologic selection, identify non-T2 therapeutic targets, and facilitate the development of personalized treatment algorithms.

### 2.5 Future challenges and opportunities in research

A critical challenge in advancing CRSmanagement lies in the standardization of immune endotyping criteria and the broader clinical adoption of these classifications. CRS is a heterogeneous inflammatory disease with multiple phenotypes and endotypes, notably distinguished by the presence or absence of nasal polyps and characterized by distinct immune pathways such as type 2 (eosinophilic) and non-type 2 inflammation (2, 138). Despite progress in understanding these immune profiles, a universally accepted, standardized immune classification system remains elusive. This lack of standardization hampers consistent diagnosis, prognosis, and selection of targeted therapies. For example, biologic agents like dupilumab (anti-IL-4Rα), mepolizumab (anti-IL-5), and omalizumab (anti-IgE) have demonstrated efficacy predominantly in type 2 CRS with nasal polyps, improving disease-specific quality of life and reducing disease burden (139). However, their application is limited by variable patient selection criteria and incomplete understanding of non-type 2 or mixed endotypes, such as those involving IL-17 pathways (140). Standardized immune endotyping would facilitate precise patient stratification, enabling personalized medicine approaches and optimizing biologic therapy use. Moreover, it would support the development of diagnostic biomarkers, such as the expression of CD11b, CD16, and CD19 immune cells, which have been correlated with clinical severity and treatment response (141). To promote clinical application, consensus guidelines integrating immune endotyping with clinical phenotypes and imaging findings are essential. This would also require training clinicians in immune profiling techniques and incorporating these into routine care pathways, ultimately improving therapeutic outcomes and reducing healthcare costs associated with refractory CRS (142).

Another significant opportunity lies in conducting large-scale, multicenter, and diverse population-based studies to validate immune endotypes and their clinical relevance across different demographic groups. CRS exhibits variability in immunopathogenesis between pediatric and adult populations, as well as among different ethnicities and geographic regions (143, 144). Pediatric CRS, for instance, often involves neutrophilic inflammation and adenoid hypertrophy, differing from the eosinophilic-dominated inflammation seen in adults with nasal polyps (145). Moreover, comorbidities such as asthma-COPD overlap (ACO) influence CRS pathology and recurrence risk, highlighting the complexity of immune interactions in different patient subsets (146). Current evidence largely derives from relatively small cohorts or single-center studies, limiting generalizability. Multicenter collaborations would enable the collection of large, heterogeneous datasets, facilitating robust phenotypic and endotypic classifications and allowing for the identification of novel biomarkers and therapeutic targets. Additionally, cross-population studies could elucidate genetic and environmental influences on CRS immune profiles, supporting the development of tailored interventions. The integration of advanced technologies such as microbiome analysis and machine learning could further enhance these efforts by uncovering complex host-microbe-immune interactions and improving predictive models for disease progression and treatment response (147, 148). Ultimately, these studies will be pivotal in translating immunological insights into effective, personalized clinical strategies for CRS management.

Biomarker compartments and validation priorities. A key translational priority is to standardize which compartments (tissue, nasal secretions, blood/plasma) are used for endotyping and how these measures are interpreted in longitudinal care. Tissue and nasal secretion biomarkers more directly reflect local epithelial–immune crosstalk, including alarmin-driven T2 programming, whereas blood/plasma biomarkers (eosinophils and IgE) are practical for follow-up but may incompletely capture mucosal heterogeneity. Future multicenter studies should therefore validate a minimal biomarker set that integrates (i) local markers of T2 activation (e.g., IL-5/IL-13 signatures and selected epithelial markers such as CST1), (ii) indicators of neutrophilic/T3 activity (e.g., IL-17-related profiles and neutrophil-associated mediators), and (iii) quantitative clinical endpoints (polyp burden, symptom scores, recurrence). Harmonizing sampling methods, assay platforms, and response thresholds will be essential to enable reproducible endotype-guided algorithms and to facilitate real-world implementation of precision therapies.

The evolving understanding of CRS immunopathology opens promising avenues for the development of novel immune-targeted therapies beyond currently available biologics. While monoclonal antibodies targeting IL-4, IL-5, and IgE have shown efficacy in type 2 CRS, the therapeutic landscape for non-type 2 and mixed endotypes remains underdeveloped (139). Emerging evidence suggests that targeting alternative pathways, such as IL-17, may benefit patients with mixed or non-type 2 inflammation, as demonstrated by the clinical improvement with secukinumab (anti-IL-17A) in refractory CRS cases (140). Future drug development should focus on these novel targets, including cytokines, chemokines, and immune cell subsets implicated in CRS pathogenesis. Additionally, combination therapies that address multiple inflammatory pathways simultaneously may enhance efficacy and reduce recurrence. The design and execution of rigorous clinical trials are essential to evaluate the safety, efficacy, and long-term outcomes of these new agents. Trials should incorporate stratification based on immune endotypes and biomarkers to identify responders accurately. Furthermore, real-world evidence studies and post-marketing surveillance will be crucial to assess broader applicability and monitor adverse events, such as cytokine release syndrome, which requires standardized definitions and management protocols (149). Advances in drug delivery systems, including topical biologics and targeted nasal sprays, may improve therapeutic concentrations at the sinonasal mucosa while minimizing systemic effects (150). Collectively, these efforts will expand the armamentarium against CRS, enabling precision medicine approaches that improve patient quality of life and reduce the burden of this chronic disease.

## 3 Conclusion

In summary, the immunoinflammatory endotyping of CRS represents a pivotal advancement that bridges basic immunology and clinical application. It challenges the traditional paradigms, enriches our comprehension of disease heterogeneity, and lays the groundwork for personalized therapeutic strategies. Continued interdisciplinary research and collaboration will be key to translating these insights into improved patient outcomes and the realization of precision medicine in CRS care.
